# Acceptability of VloV, a Mobile App Developed in Latin America for People with Substance Use Disorder among an Intensive Outpatient Treatment

**DOI:** 10.62641/aep.v53i2.1701

**Published:** 2025-03-05

**Authors:** Diana Milena Berrio Cuartas, Carola Cassinelli, Luciana Noemi Garcia, Federico Pavlovsky

**Affiliations:** ^1^Dispositivo Pavlovsky, C1425EFD Ciudad Autónoma de Buenos Aires, Argentina; ^2^Instituto Multidisciplinario de Investigaciones en Patologías Pediátricas (IMIPP) CONICET–GCBA, Hospital de Niños Ricardo Gutierrez, C1425EFD Ciudad Autónoma de Buenos Aires, Argentina

**Keywords:** mobile applications, digital therapeutic tools, addiction, therapeutic alliance

## Abstract

**Background:**

Digital therapeutic tools seem to be helpful for substance use disorders (SUD), but there are few studies in Latin America about this approach. Our group of therapists developed VloV (an abbreviation for Pavlovsky), a mobile app that attempts to digitize practical tools along with strengthening the therapeutic alliance and user practice.

**Method:**

We conducted a mixed-method pilot study between August 2021 and January 2022 to collect data about the patient experience using VloV and the therapeutic alliance among 23 patients. VloV is a Spanish mobile app available for free that focuses on SUD and covers therapeutic elements and tools from an intensive outpatient treatment program. A monthly PDF report containing the patient’s daily interactions is generated and can be shared via e-mail with the therapist for follow-up. We run three questionnaires (Q), Q1 regarding technology use indications Q2 to review content information and the utility of the different elements of VloV, and Q3 to collect qualitative data about participants’ experiences and perceptions. Several aspects of the therapeutic alliance were evaluated using the patient version of the Working Alliance Inventory in its short version (WAI-S-P). Several aspects of the therapeutic alliance were assessed using the patient version of the Working Alliance Inventory in its short version (WAI-S-P). The level of agreement between the raters—provider and monthly VloV reports—was calculated for treatment variables in patients and their mood state records. For some sub-analysis, patients were divided into two categories, those who have a daily app’s use and those who have a weekly or sporadic use.

**Results:**

Patient characteristics were similar to the statistics of the treatment center data, including the dropout rate; only 15 out of 23 (65.2%) patients completed the 12 weeks of the pilot study. Participants reported daily use of the technology, but only 5.0% searched for health topic information on the web. Patients expressed positive feedback by using the app and found some functional aspects in VloV that contributed to their treatments and self-care as mood scale record, money earned display, sobriety calculator, and treatment skill functions. However, the “red button” function, which allows the patient to ask for help, was not found to be of much use. We found a correlation between the frequency app’s use and a higher accuracy in the provider register of treatment related to variables. Although working alliance therapeutic scores were mainly high and non-differences were found.

**Conclusion:**

This is the first study on a mobile application for SUD developed in our region, and although it is only a preliminary study, it pointed out important lessons about incorporating digital therapeutic tools into mental health treatment in an intensive outpatient treatments (IOT) setting.

**Clinical Trial Registration:**

Sistema Integrado de Información Sanitaria Argentino (IS004799).

## Introduction

Over 2% of the world population has a substance use disorder (SUD). In Latin America, only 1 of 11 patients received treatment for SUD according to the United Nations Office on Drugs and Crime—UNODC [1]. A recent epidemiology study of mental health reported a 10.4% for life prevalence of SUD in Argentina, with rates of 41.6% in access to treatment and 2.6% for early treatment [2].

Following principles such as respecting patient autonomy and preventing disruption of social ties, intensive outpatient treatments (IOT) for SUD patients have grown [3]. Nevertheless, primary challenges persist in traditional IOT, including relapse prevention and barriers to access, such as high costs, geographic dispersion, limited schedules, and the absence of tailored peer support [4].

The health crisis associated with coro-navirus disease 19 (COVID-19) pandemic produced harmful patterns of drug use but also impulse remotely-provided treatments and multiple types of digital tools for mental health that are now an established part of the digital health landscape [5]. Evidence-based Digital Therapeutic Tools (DTT) offer promising solutions to address these challenges, as they are portable, capable of receiving and transmitting data, and provide healthcare providers with the unique opportunity to connect with hard-to-reach populations [6].

Little is known about the use of digital platforms among patients attending outpatient substance use disorder treatment programs [5, 7]. Although there are international reports that pointed out a possible contribution of DTTs in IOT there are, as far as we know, no studies in Latin America about digital health tools approaches to the management of SUD [8, 9].

As an IOT program based in Argentina, in 2021 we explore expanding accessibility strategies, and in 2022 the clinical staff of our group initiated the development of VloV (an abbreviation for Pavlovsky), a Spanish free mobile app for SUD patients, to digitize practical tools, strengthen the therapeutic alliance and the provider practice [10, 11]. VloV emerges as an option for individuals who do not require an inpatient facility but need a more intensive approach than traditional treatment. The program promotes strategies based on cognitive behavioural, motivational skills, and dialectical behaviour therapy integrated under an intensive group therapy setting. A set of tools (daily phone contact with therapist, substance use diary, list of reasons for not consuming, differential reinforcement of incompatible behaviour, daily mood state log) were elaborated under the Patient-Centered Care model related to comprehensive care, individual psychotherapy, shared decision making and therapeutic alliance [12]. These tools were developed and independently applied in clinical practice since 2010, where they were perceived as beneficial for patient progress. Subsequently, they were digitized and incorporated into the VloV as features within the application for daily, continuous, and integrated use by patients. Data collection was initiated for the analysis of their effectiveness.

Concurrently with the app release, we conducted a pilot study to collect quantitative and qualitative data about VloV’ impact on patients and therapists over a SUD treatment in an IOT setting.

## Materials & Methods

### Participants 

Eligible individuals were 18 years old or above that could write and understand the Spanish language; had a diagnosis of SUD as determined by the criteria of the Diagnostic and Statistical Manual [13]; initiated treatment in the IOT program of the Dispositivo Pavlovsky and were able to consent to receive treatment. The number of patients that fulfilled the inclusion criteria were 23, during the 3-months of the pilot study only 15 patients completed treatment. Through the 3-months of the pilot study, eight patients’ voluntary withdrawal from treatment on weeks: 2, 3, 4, 5, 8, 9, 10, and 11 respectively. The study was registered (IS004799, the agency is Sistema Integrado de Información Sanitaria Argentino, the link is https://sisa.msal.gov.ar/sisa/#sisa), approved and conducted in accordance with the Biomedical Research Ethics Committee of the Institute of Translational Medicine Research (IATIMET), University of Buenos Aires (approval number: 9-11-2021) and adhered to the tenets of the Declaration of Helsinki. All participants provided written informed consent.

A mixed method pilot study was conducted on these patients to evaluate acceptability and whether the use of VloV mobile app impacted the working alliance with their therapist. Study recruitment took place in the Dispositivo Pavlovsky, an IOT private institution, between August 2021 and January 2022. The study was conducted among patients in a one year-IOT setting at Buenos Aires, Argentina, during the first 12 weeks of the treatment.

### VloV Structure

**Fig. 1.**
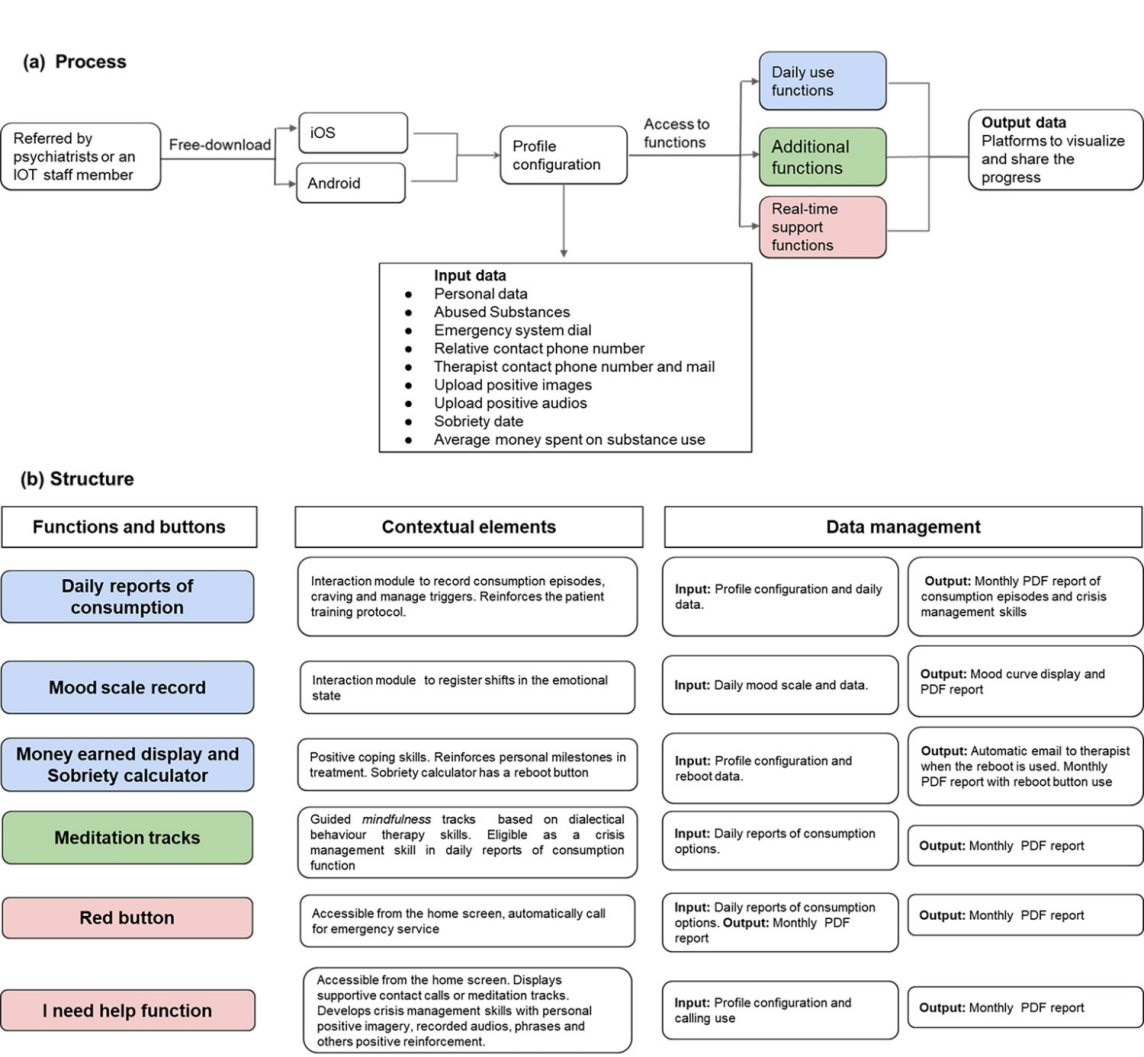
**Process and structure of VloV**. (a) Typical pathway for VloV’s use. (b) Components, rationale, and data management of the structure functions of VloV. Daily use functions are colored in blue, while additional functions and real-time support functions are shown in green and pink (respectively). Input data: information provided to the app software during the profile configuration and daily use. Output data: processed data presented through the interface.

**Fig. 2.**
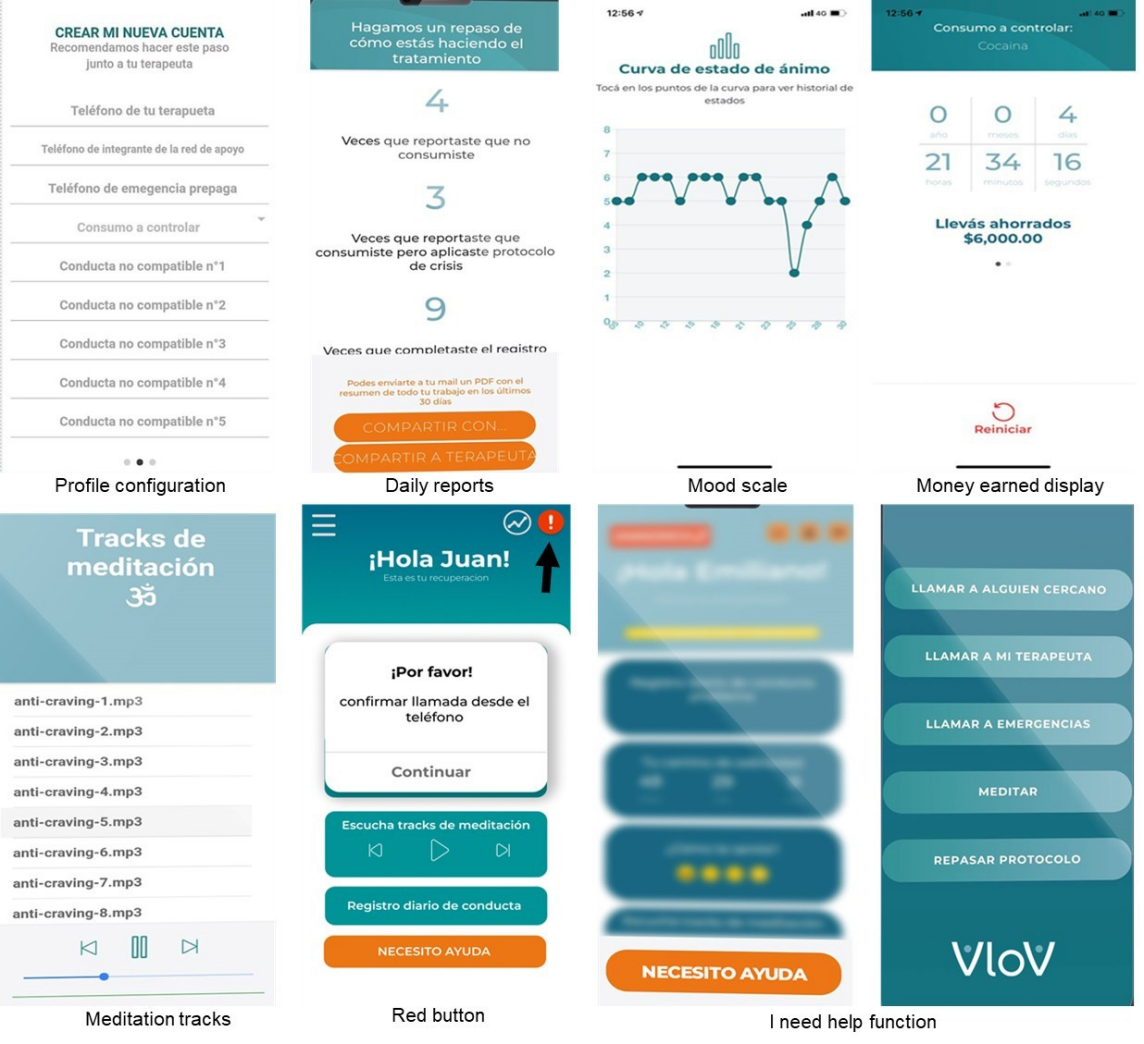
**Representative screenshots of VloV in use**. An arrow points out the function to the red button function on the home screen.

VloV is a hybrid mobile app and therefore can be used across all mobile devices. The app is available for free to download in both iOS and Android devices [14]. A schematic process and structure of VloV with its rationale are described (Fig. 1); the app includes: (a) interaction modules for substance use diary, (b) positive coping skills (sobriety calculator and money earned display), (c) emotion monitoring, (d) meditation tracks to manage craving crises, difficulties in emotion regulation, including anxiety (e) a “red button” function that initiates a call to the emergency system along with a warning alert to the therapist staff and (f) a module of real-time support (“I need help”) to manage craving or consumption crises with customized positive images, audio, and data or initiating a call to a designated relative, the therapist or ambulance. A monthly PDF report containing the patient’s daily interaction with the functions is generated and can be shared via e-mail with the therapist for follow-up. Representative screenshots of VloV in use are shown in Fig. 2.

### Other Instruments

Demographic variables included, primary substance use disorder, and relevant clinical data was recorded. We administered three questionnaires. Questionnaire one (Q1) contains an adapted version of a technology use questionnaire [15]. Questionnaire two (Q2) consists of a remotely administered questionnaire, designed for this study, that uses a Likert scale of 0–5 (0 = lowest; 5 = highest) to measure the content information and the utility of the different modules of VloV. Questionnaire three (Q3) was a semi-structured interview designed for this study to collect personal experience of the patient about the use of VloV. The interview lasted between 30 and 60 minutes, was conducted in person and registered in notes.

The Therapeutic Alliance was studied using the Spanish version of the patient form of the Working Alliance Inventory in its short version (WAI-S-P) (Cronbach’s alpha coefficients reported for overall measures and their corresponding subscales ≥0.86) [16].

For some sub-analysis, patients were divided into two categories, those who have a daily app’s use and those who have a weekly (an average usage of three times per week) or sporadic (an average usage of one or less per week) use.

### Procedure

After participant consent, VloV was downloaded. Participants completed self-report measures and different assessments (WAI-S, Q1, Q2, Q3) during the 12 weeks. Participants’ data regarding VloV use was collected along with a technical counselling intervention at 2, 8 and 12 weeks from the enrolment time. During the visit of the 2-week of treatment patients complete the Q1, at the 4-week interview patients complete the Q2. At the 12- week the patients complete the WAIS-S-P scale and have the interview for the Q3 asses.

In this pilot study, the app was used uniquely by participants and not in interaction with their therapy providers. The therapist’s assignment was following the regular procedures of the institution and prior to the patient’s invitation to study. A monthly report of the abstinence status and progression of the patient was obtained from the therapist, which permits the differences in the app report and the provider register of abstinence status and commitment to the treatment. We defined study completion as staying in treatment through the 12 weeks of the pilot study.

### Data Analysis

The study used descriptive statistics to summarise the demographic information (mean, standard deviation, range). Non-parametric tests such as the Wilcoxon-Mann-Whitney test and Kruskal Wallis were applied to compare groups, while Spearman correlation was calculated under the non-normality data. The comparison of proportions on the technology utilization questionnaire as well as some characteristics between patients with different app’ use patterns was administered using Fisher exact test. The level of agreement in mood state between the therapist report and VloV’ document was calculated with Cohen’s kappa coefficient. All analyses were conducted using InfoStat (versión 2020, Universidad Nacional de Córdoba, Córdoba, Argentina) (https://www.infostat.com.ar/).

## Results

### Patients 

The demographic data of the 23 patients enrolled for this pilot study are described in Table 1. 69.6% of the participants were young males and had alcohol use disorder as their main diagnosis followed by cocaine use disorder. 78.3% were single at the time of the study. 39.1% of the participants had a high academic level and 69.6% were employed. Notably, 4 (17.4%) patients have a diagnosis of borderline personality disorders. Other relevant antecedents of the study participants are also described in Table 1 and are similar to the statistics of the treatment center data, including the dropout rate [11]. During the 3-months of the pilot study, the rate of the dropout was 34.8%, and eight patients’ voluntary withdrawal from treatment on weeks: 2, 3, 4, 5, 8, 9, 10, and 11 respectively; while 20 patients completed the Q1 and Q2 surveys, only 15 patients completed treatment and the Q3 survey.

**Table 1.** **Demographics data**.

Age (years): (Q1–Q3)		34 (26–43)
Gender: N (%)		
	Male	16 (69.6%)
	Female	7 (30.4%)
Substance use: N (%) #		
	Alcohol diagnosis	12 (52.2%)
	Cocaine diagnosis	11 (47.8%)
	Cannabis diagnosis	2 (8.7%)
	Other drug diagnosis	2 (8.7%)
Marital status: N (%)		
	Couple	5 (21.7%)
	Single	18 (78.3%)
Employment: N (%)		
	Employed	16 (69.6%)
	Unemployed	7 (30.4%)
Education N (%)		
	Less than high school diploma	2 (8.7%)
	High school diploma	12 (52.2%)
	More than high school diploma	9 (39.1%)
Related variables N (%)		
	History of sexual abuse (yes)	2 (8.7%)
	Legal problems (yes)	6 (26.1%)
	Previous overdose (yes)	7 (30.5%)
	Attempted suicide history (yes)	2 (8.7%)
	Physical health problem (yes)	0 (0.0%)
	Inpatient intervention in the past 3 months (yes)	1 (4.4%)

# Some patients have more than one substance use disorder.

### Surveys Analysis

#### Technology Utilisation Results

**Fig. 3.**
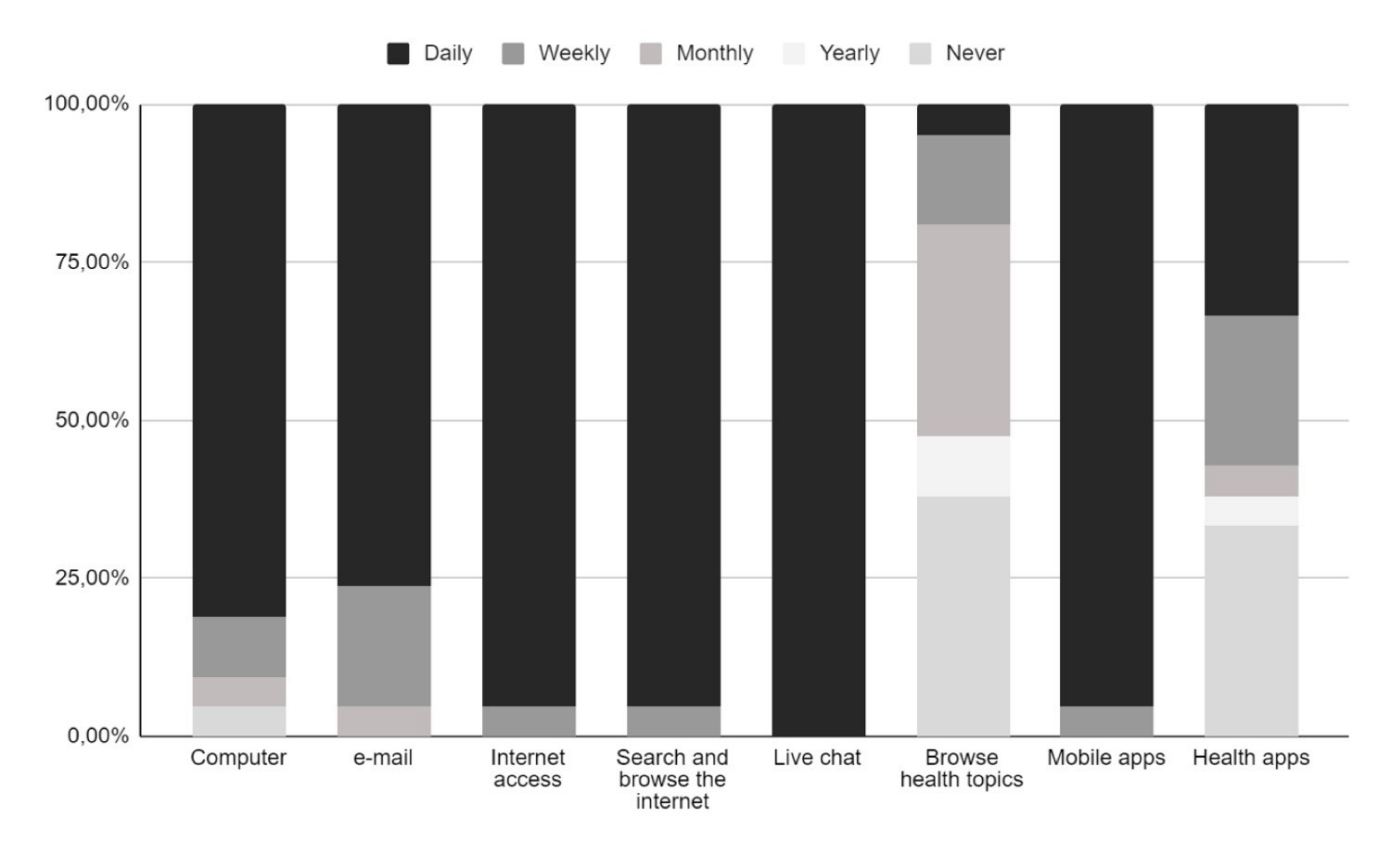
**Technology utilization questionnaire results**. The figure expresses percentages of responses per technology.

We initially studied the technology utilization patterns of the patient using an adapted version of a technology use questionnaire (Q1) [15]. The median (Q1–Q3) of participants who reported daily use of the technology was 18 (13–19) (Fig. 3). Only five patients have another frequency of use. While specific health information searches and apps were monthly, yearly, or never used. The low searches of health topic information on the web and the low-frequency use of health apps may reflect low exposure to recovery information and low previous interest in receiving health care support. There was a significant difference in the proportion of live chat and search of health topic information on the daily use utilization pattern (100.0% vs 5.0%, *p* < 0.001).

### Evaluation of the Content Information and Utility of the Different Modules of VloV

Across the functions (Table 2), the percentage of responses distributed in higher levels of appropriateness were: mood scale record, money earned display, sobriety calculator, and treatment skills review. While meditation tracks, “red button”—utility and content—obtained lower scores. Profile content and daily consumption records were scored mostly neutral.

**Table 2.** **Content information and utility score obtained in the structure of VloV**.

Structure/scale	Inappropriate	Slightly inappropriate	Neutral	Appropriate	Absolute appropriate
VloV’ information provided	0	5	35	15	45
Red button’ information	5	10	50	20	15
Treatment protocol review	5	0	35	15	45
Profile data	0	5	40	25	30
Daily report of consumption	5	0	40	15	40
Sobriety calculator and reboot button	0	10	20	35	35
Money earned display	10	5	15	25	45
Mood scale records	5	0	25	5	65
Meditation tracks content	0	5	60	15	20
Meditation tracks utility	5	15	45	15	20
Red button’ utility	0	15	40	20	25

Information is described as the percentage of responses (n = 20).

### Personal Experience of the Patient with the Use of VloV

A semi-structured interview (Q3) was conducted with 15 patients to gather their personal experience about the use of VloV. The interview began with the facilitator asking: *Do you recommend this app to anyone interested in SUD recovery?* Almost all the patients (93.4%) replied affirmatively. One patient said, “The app is user friendly”. Another shared, “It [VloV] keeps you connected to your treatment, reflects your sobriety and your mood perspective”. A different patient noted: “It seems to me a good way to reach patients who still have difficulties forming a connection with the treatment or overcoming fear or shame, to be honest with professionals”. Some participants suggested that VloV could be recommended as a tool for someone in a similar setting but not as a standalone treatment.

The next question was: *Has the use of the VloV app impacted your current treatment care?* Responses were mixed, with only 40.0% answered affirmatively. Participants who felt that VloV had not impact on their treatment cited timing as a factor: “The use of VloV with individual therapy is somewhat disconnected; the app makes me reflect on the day-to-day”. Others mentioned conflicted personal reasons, such as, “My mood varies a lot during the day, and the app does not help to register it”.

When asked, *How would you describe the relevance of VloV in registering your situation in the treatment? *a high percentage (73.4%) of the participants felt that VloV aids in their adherence. One patient said, “It [VloV] helps me record my mood swings, when I notice changes, I am more alert, it makes me reflect, and change my attitude”. Others frequently mentioned the functional tools that enhance positive coping skills (See more detail in Table 3). Regarding the treatment we asked: *Are there aspects of the app that contributed to your effectiveness in treatment?* Again, almost all the patients (86.7%) responded positively. They said: “The treatment is being effective for me, and everything that connects me to the treatment is helping me”. “Yes, it helps me record how I am on a daily basis and ask myself why I’m feeling this way?”. “[It contributed] to record the restarts and the aspects that needed to be worked on”.

**Table 3.** **Experiences, perceptions and opinions of the participant about the app’s utility and impact**.

VloV’ contributions to providing		Areas of suggested improvements	
Mood scale record and sobriety calculator	Profile and other functions	Red button	Others
“It [VloV] helps to record mood status[.] that motives such as money [saved] and the sobriety calculator are described [in it,] which helps to better appreciate these resources”	“I use a photo that is not mine, but it works for me, it is a scene from a TV show”	“I found it easier to make a call, it doesn’t seem as direct from the app.”	“It seems to me that abstinence for different substances is not adequately contemplated”
“The mood scale helps you evaluate how you have been over the previous days, such as changes during the transition between therapist”	“[VloV] suggests reasons for not consuming. I mainly use non-compatible behaviours [function]”	“If needed, I call my therapist or engage in a non-compliant behaviour”	
“It helps me be more specific about mood state, clarifies craving emotions and mood state”	“The tool I like the most is the meditation, although I don’t use it very often”	“I am more accustomed to calling on the phone or looking for an external method to ask for help. I feel that I did not use of the button correctly”	

Other questions addressed the use of the app’s functions. 80.0% completed all aspects of the profile (which included motivation audio and images that could later be used for coping skills). Only four patients reported using the “red button” function and they get instant feedback from their therapists. The remaining 11 stated that in an emergency, they would prefer to call their therapist or a family member. Notably, one patient used the warning alert for another urgent communication need and also received instant feedback from the therapists.

We also asked: *Do you choose to share VloV’ report with someone, and if so to whom? *Responses were divided, with only 60.0% sharing the app’s report, all with family members. Participants emphasized: “I shared it with my family because I needed to tell them how I felt”, and “Yes, with my parents because they were curious about how the app works”. Finally, we asked if they noticed any change in their personal experience with the mobile phone. Only three patients felt a change, with one noting, “I established a routine to use the app on the mobile every day.”. Notably, one patient who relapsed and dropped out of treatment within the first month traded the mobile phone for drugs.

Participants emphasized two elements of the app that were particularly useful and impactful: the mood scale, money saved and sobriety calculator while many consider the “red button” feature to be less useful (Table 3).

Despite these useful elements and positive impacts, participants made it clear that the app’s utility was primarily as an additional tool of their current treatment rather than a standalone solution.

### Therapist Reports and VloV’ Report Results

An analysis of the differences in the app report and the provider’s monthly record of three categorical variables related to the treatment and evolution were conducted: use of the app (Yes/No), abstinence status (Yes/No), use of the therapeutic tools (Yes/No). A concordance ratio was calculated for these variables on those measured in the reports of 10 therapists about the 15 patients who completed the three-months period. The median (Q1–Q3) value of the score obtained was 0.77 (0.66–0.88).

### Analysis of the Mood State of the Patients

Another assessment related to the data provided for VloV and the data on the charter of the patients was the function called mood scale records. We calculated the agreement in the information provided along the 3 months of the pilot study using the Cohen’s kappa coefficient of agreement in mood state between provider and VloV reports. The median (Q1–Q3) value was 0.5 (0.20–1), which could be interpreted as moderate agreement.

### Working Alliance Results

At 12 weeks of initiating the treatment, the WAI-S-P was administered to 15 patients. High values were obtained regarding the higher possible scores (28 in subscale and 84 in total score). Median (Q1–Q3) for total score was: 78 (75–79), while for the subscales bond was the item with the highest values: 27 (24–28) compared to goal: 26 (23–27) and task: 25 (24–27).

Although the app was used uniquely by participants and not in interaction with their therapy providers in this initial report, we wanted to find out if the pattern of use determined in the report of the app can be related to the alliance between patient and provider.

### Subgroup Analysis Based on Usage Frequency and Follow-up

We qualified patients in two categories, those who have a daily app’s use and those who have a weekly or sporadic use in order to perform a secondary data analysis of the therapist reports and working alliance questionnaire (Table 4). No significant differences in all demographic data were determined, the analysis is summarised on Table 4.

**Table 4.** **Demographic of subgroup analysis and correlation**.

Summary of demographic data		Patients that have a daily use of the app (n = 8)	Patients that have a weekly or sporadic frequency use of the app (n = 7)	Statistical value	*p*
Age: median (Q1–Q3)		39 (33–42)	27 (24–37)	W: 45.0	*p* = 0.21
Gender: female/male		2/6	3/4		*p* = 0.60
Substance use:					
	Alcohol diagnosis	2	1		*p* = 0.99
	Cocaine diagnosis	2	3		*p* = 0.60
	Other drug diagnosis	4	3		*p* = 0.99
Marital status: couple		1	1		*p* = 0.99
Employment: yes		5	4		*p* = 0.99
Education: high school diploma		4	4		*p* = 0.99
Related variables					
	History of sexual abuse	1	1		*p* = 0.99
	Legal problems	3	3		*p* = 0.57
	Previous overdose	3	4		*p* = 0.60
	Attempted suicide history	1	1		*p* = 0.99
	Inpatient intervention in the past 3 months	0	1		*p* = 0.47
Concordance ratio between reports: median (Q1–Q3)		0.77 (0.66–0.88)	0.72 (0.66–0.77)	W: 54.0	*p* = 0.09

W, W-Value from Wilcoxon-Mann-Whitney test.

Although there were no statistical differences on the concordance ratio between therapist and VloV reports (Table 4), there was a moderate correlation between the congruency of the reports and the weekly frequency app’s use (*p* = 0.042, R^2^ = 0.50).

In both (subscale and total dimension) of WAI-S-P scores no significant difference was found between median scores by Mann Whitney test. In addition, Cohen’s kappa coefficients were similar (Table 5).

**Table 5.** **Statistical analysis of the WAI-S-P (total and subscales) according VloV’ use patterns**.

WAI-S-P scale	WAI-S-P score among patients that have a daily use of the app (n = 8). Median (Q1–Q3)	WAI-S-P score among patients that have a weekly or sporadic frequency use of the app (n = 7). Median (Q1–Q3)	Statistical value	*p*
WAI-S-P bond	26.5 (24–27)	28 (24–28)	W: 62.0	*p* = 0.51
WAI-S-P task	25 (24–27)	27 (22–28)	W: 59.5	*p* = 0.71
WAI-S-P goal	25.5 (23–26)	26 (21–27)	W: 59.5	*p* = 0.70
WAI-S-P total	78 (75–79)	82 (58–82)	W: 61.0	*p* = 0.57
Cohen’ kappa coefficient	0.25 (0.23–0.60)	0.28 (0.23–0.43)	W: 34.5	*p* = 0.40

W, W-Value from Wilcoxon-Mann-Whitney test; WAI-S-P, Working Alliance Inventory in its short version.

After completion of the study, data collection was discontinued, some patients continued to use VloV along with the newly admitted patients, but not systematised data were collected. At a 6- months follow-up, 7/15 (46.7%) patients remained on treatment. 4/8 (50.0%) were on daily use during the pilot study and 3/7 (42.9%) were on the sporadic use profile. We are currently designing an update of VloV along with a version to family members or relatives of a person being treated for SUD.

## Discussion

A significant proportion of digital health apps released are eventually removed from the market. Constant update and patient perception are crucial to overcoming the barriers to adoption [5]. In this preliminary pilot study, we collected quantitative and qualitative data on the impact of VloV on patients and therapists to evaluate its performance. VloV was generally found acceptable and feasible, although some functions need improvement and its specific contribution to the therapeutic alliance warrants further study.

Participants in the pilot study demonstrated sporadic use of specific health information searches and health apps. Nevertheless, the low exposure to recovery information did not interfere in the acceptance of VloV. A recent study reported that nearly half of participants in SUD treatment in an IOT setting accessed content on social media that triggered substance cravings, but they were generally receptive to using relapse prevention apps and text messaging interventions [7].

In the study, 93.4% of the patients said they would recommend this app to another patient in an IOT setting, and a high percentage felt that using the app contributed to the treatment adherence. This observation has been described for the DTT for SUD based on the digital Therapeutic Education System, along with a correlation between patient engagement and the probability of abstinence at 9–12 weeks of treatment [9]. Despite these useful elements, participants made it clear that the app’s utility was mainly as an additional tool in their current treatment and not its own.

Patients found some functional aspects of VloV that contributed to their treatments and self-care: mood scale record, money earned display, sobriety calculator and treatment skill functions, while “red button” function was not found to be of much use. This demonstrates the challenge of how a digital aid could be adjusted based on patient population and setting, as in another study, providers suggested incorporating an alert option for emergency [8].

This pilot study has potential limitations. The app was used exclusively by participants and not in interaction with their therapy providers to establish a baseline contribution of the app’s use, and the small sample size restricted the generalizability of the results. In our preliminary study we found a correlation between the frequency of app use and greater accuracy in the provider’s record of treatment-related variables. Also, the level of agreement in the patient’s mood state between the medical record and the daily mood data on VloV varied considerably between patients. However, working alliance therapeutic scores were generally high, and non-differences were detected between patients with different app use profiles. A study on reasons for premature termination of SUD treatment from client and clinician perspectives noted that important dropout reasons could be influenced by a lack of early therapeutic alliance development [17]. The study by Palmer *et al*. [17] also highlights that therapists may consider utilizing new techniques to rapidly build and maintain the therapeutic alliance. The data recorded in VloV could be crucial for detecting barriers to acquiring therapeutic tools, contingency management and mood state. In this context, in-person and mobile treatments can complement each other, potentially improving treatment attachment.

The lesson to be learned from these experiences is that patients perceive VloV as another tool of their treatment, effective in concordance with the program and not on its own. This has been highlighted in a recent report that compared three apps for SUD and concluded that they do not prove their value and effectiveness in delivering benefits directly, as established methods have [18]. Additionally, some patients share the app report with their relatives. There is cumulative evidence about the efficacy of family-based interventions for SUD [19], and relatives were not initially contemplated in the app. This may suggest a need for an update of VloV to open new communication channels and also provide support tools to them.

## Conclusion

This is the first study on a mobile application for SUD developed in our region, and although it is only a preliminary study, it pointed out important lessons about incorporating digital therapeutic tools into mental health treatment in an IOT setting. Further studies are needed on aspects such as the working alliance, the inclusion of a module for relatives, and the durability of the clinical effects on patients.

## Availability of Data and Materials

The data that support the findings of this study are available from the corresponding author, upon reasonable request.
